# An exploration of caregiver burden for children with nodding syndrome (lucluc) in Northern Uganda

**DOI:** 10.1186/s12888-016-0955-x

**Published:** 2016-07-22

**Authors:** Janet Nakigudde, Byamah Brian Mutamba, William Bazeyo, Seggane Musisi, Okello James

**Affiliations:** Department of Psychiatry, Makerere College of Health Sciences, P.O. Box 7072, Kampala, Uganda; Butabika National Mental Referral Hospital, Kampala, Uganda; School of Public Health, Makerere College of Health Sciences, Kampala, Uganda; Gulu Medical School, Kampala, Uganda

**Keywords:** Nodding syndrome, Caregiver, Caregiver burden, Northern Uganda

## Abstract

**Background:**

Caregivers of patients with chronic illnesses are often uncompensated for work that is physically demanding, time consuming and emotionally and economically draining. This is particularly true for caregivers of children with nodding syndrome, an emergent neurological disorder of unknown etiology in resource poor settings in Africa.

We aimed to explore perceptions of caregivers regarding challenges that a typical caregiver faces when caring for a child with nodding syndrome.

**Methods:**

We used a qualitative exploratory study design with focus group discussions and in-depth interviews to collect data. We analyzed data using the qualitative analysis software package of NVivo and thematic query building.

**Results:**

Emergent themes centered on burden of care with emotional agony as the most prominent. Subthemes reflecting the burden of care giving included child and caregiver safety concerns, burnout, social isolation and rejection, and homicidal ideation. Caregivers also complained of physical and financial constraints associated with the care of children with nodding syndrome.

**Conclusions:**

The findings point to a high burden of care for caregivers of children with nodding syndrome and suggests the need to incorporate community-based psychosocial and mental health care services for the caregivers of affected children into the national health system response.

## Background

Nodding syndrome has been described in areas that have experienced adversities such as the prolonged wars in northern Uganda and southern Sudan [1]. These are resource poor settings with limited access to primary health care services. The syndrome, initially reported in Tanzania [2, 3], has subsequently been reported in Liberia [3, 4], the republic of south Sudan [5, 6], and western and northern Uganda [1, 6]. More than 3000 children have been affected by nodding syndrome in post-war northern Uganda that faced chronic adversity, internal displacement and disrupted social networks following more than 20 years of armed conflict [1].

Nodding syndrome is described as a chronic non-communicable, epileptic disorder of unknown origin that afflicts children and adolescents ranging in age from 3 to 18 years [1]. The key symptom is a repetitive dropping forward of the head due to a loss of neck muscle tone at a frequency ranging from 5–20 head-nodding episodes per minute; this is sometimes associated with loss of muscle tone in the trunk and upper extremities [2]. Early symptoms of nodding syndrome are characterized by head nodding with or without other seizure types, hence the name.

Head nodding usually occurs at feeding times and during cold weather. In the later stages of the illness, children may present with cognitive deficits, malnutrition, behavioral problems [7], delayed sexual and physical growth, wasting or stunting and psychiatric difficulties [1]. For a child to be diagnosed with nodding syndrome, he or she must have been previously healthy without any physical or psychological symptoms. As symptoms progress, they become debilitating, eventually causing cognitive, motor, behavioral and social impairments [8]. Studies have shown that often times rather than one child in a family being afflicted, more children in a household may sequentially become afflicted by the disorder at different times compounding the family distress and burden of care.

Because of the associated mental and neurological deficits, caregivers may face stigma and discrimination [9]. Dealing with stigma and other negative care giving experiences overwhelm the family’s available resources. Community surveys and experiences from the nodding syndrome response within the health system in Uganda suggest that the majority of children with nodding syndrome are treated in primary care, without admission to a health facility. When admitted, because of limited resources, acutely ill children are often discharged before they are fully recovered, with further management left to family and close relatives who provide a more supportive role [10].

Given the circumstances, the family caregiver experience has been one that is emotionally and physically draining. Despite these major stresses experienced by caregivers, few studies have explored these challenges in this particular population afflicted by this particular chronic disorder.

Caregiver experience has been described differently by various researchers [11]. To illustrate this, the bio psychosocial consequences of caregiving have been described as caregiver strain [12], caregiver stress [13–15], caregiver appraisal [15, 16], caregiver burden [17] and several other illustrations. Our focus of discussion regarding caregiver experiences is centered on caregiver burden. Caregiver burden has received considerable emphasis in the literature on the social experience of caregiving children with disabling illnesses, such as nodding syndrome. Little attention has been paid, however, to the nature of the burdens perceived.

Caregiver burden has been described as the distress a caregiver experiences that is as a result of the care recipient’s physical dependence and mental incapacity [18]. This concept of burden has been broadened to include two types of distress experienced by the caregiver: that due to his or her provision of care and distress due to the impact that care giving is having on one’s life. To further understand caregiver burden, it is important to critically examine how various researchers have described this concept. In Global Health [19], caregiver burden is described as the negative effects to the family as a result of looking after a care recipient. These negative effects are perceived to be a mediating factor of the patient’s disability along with the negative consequences of the care giving. Other researchers [20] have dichotomized caregiver burden splitting it into objective and subjective dimensions. The objective dimension of burden is one that includes events and activities related to the negative care giving experiences while the subjective burden of care giving relates to feelings aroused in caregivers as they are carrying out their caregiver roles. There are researchers who describe objective burden as the extent of changes in a caregiver’s life in order to incorporate the patient’s needs and subjective burden as the caregiver’s attitude or emotional reactions to the care giving experiences [21]. Another study by Platt, further reports that objective burden is measurable and observable while subjective burden is about the caregiver’s personal feelings [22].

Later studies on caregiver burden have not only looked at objective and subjective dimensions, but have also isolated the variables that contribute to caregiver burden. Caregiver burden is additionally described as the “extent to which caregivers perceive their emotional, physical health, social life and financial status that is resultant of taking on the role of care giving” [16]. Similarly, Kosberg identified variables in burden of care to be physical, psychological or emotional, social and financial problems that caregivers for an impaired or disabled member of the family experience [23].

More recent studies have focused on the recurring attributes of burden of care identified in previous research [24]. Walker and Avant have theorized the burden of care as including perception, as well as a multidimensional phenomenon characterized by dynamic change and overload [25]. More specifically, a caregiver’s perceived burden of care is dependent on the interpretation he or she makes of the demands of the patient and the available resources. In quantitative studies of the multidimensional nature of burden of care, factor analyses have revealed high factor loadings based on the physical, financial, psychological and social attributes [26]. Explorations of the dynamic change in burden of care, have pointed out that burden changes with demands over time which may be due to the increasing disability or impairment of the patient. The demands that are resultant of the increasing disability and or impairment of the patient lead to the overload attribute which essentially is a result from an imbalance between the perceived demands of the patient, and the caregiver perceived resources. As a result, many caregivers suffer from psychological distress like depression [27].

Against this background of caregiver burden attributes, is a wealth of predisposing factors that are influential long before the caregiver begins to perceive the burden of care giving [28]. These factors increase the risk and vulnerability for caregiver burden and they include gender, socioeconomic status, race and culture, health and psychological factors. Regarding gender, it has been shown that the burden of care giving is experienced differently by males in comparison to females, with females taking on the multiple roles of mother, primary emotional supporters and household managers. Females are more likely than males to be caregivers and because women have been socialized into the role of care giving, they perceive their burden to be greater than that of male caregivers, who focus on task accomplishment in their role as caregivers [21, 27–29].

Socioeconomic status and caregiver health have also been found to increase vulnerability to caregiver burden. The amount of income available determines whether services that a caregiver needs for the patient can be purchased to alleviate the burden of care giving [27]. Apart from family finances, the physical and mental state of the caregiver is important. Specifically, a caregiver who is physically or mentally unwell is likely to perceive a higher level of burden of care than one who is in overall good health [30].

Although psychological factors related to burden of care have not been intensely investigated, some research has found a caregiver’s sense of obligation and responsibility to be correlated to burden [29]. The nature of the relationship, including affection that the caregiver has for the care recipient has also been found to be correlated to burden of care [29].

There is evidence that caregivers of persons with chronic illnesses suffer from significant stresses and high levels of burden [20, 30, 31]. The caregiver is usually a relative and the care given is most often continuous. The caregiver often has additional responsibilities in the family and the care is given because of emotional bonding, duty, guilt and/or the lack of other available services in the community [32]. The few studies that relate to caregiver burden in northern Uganda do not distinguish between the individual stressors that families may experience and the resulting strain that these difficulties produce [33].

In sum, there is no consensus on what constitutes burden and its measurement is limited. Different variables in subjective and objective burden might have different levels of impact on different caregivers, and the outcome of that distress may not be the same for all individuals. The key dimensions of burden that have been measured in the literature [34], include: (a) symptom-specific burden; impact of the disability associated with the illness itself, both in terms of demands for assistance and supervision, and regarding the potential stigma associated with the illness, (b) social burden; impact on family and other social relationships, (c) emotional burden; impact on mental and emotional well-being and (4) financial burden; impact on work and the general financial costs of care-giving [35]. In northern Uganda, studies exploring the nature and effect of caregiver burden are limited in scope. We sought to explore challenges in the care giving experiences of caregivers of children with nodding syndrome, with a focus on all four dimensions of caregiver burden.

### Study setting: Northern Uganda

This study investigated caregiver burden in Uganda, a low income sub-Saharan country with an income per capita of US $ 547. The income per capita in the study region is even lower than that of the general Ugandan population due to an insurgence that lasted twenty-one years. The literacy rate in the country stands at 81 % for males and 61 % for females [36] but is thought to be much lower in the particular region where the study was conducted as a result of the insurgence. During the conflict, people were institutionalized in internally displaced people (IDP) camps, and on returning to their communities, many realized that everything had changed including the land tenure system on which their livelihood depended. Some of them were thus rendered landless: considering that the main source of livelihood was cattle keeping and crop farming, this created major difficulties for some families with respect to reconstructing their lives. The widespread killings which characterized the conflict resulted in the destruction of the social support fabric, leading to a higher than usual prevalence rate of trauma, grief, and depression with manifestations including suicide, learned helplessness and substance abuse to mention but a few of the effects [37]. Thus, the aim of this study was to explore the challenges that caregivers of children with nodding syndrome are faced with in their daily care of affected children.

## Methods

We used an exploratory research design that included qualitative data collection and analysis methods. Focus group discussions and in-depth interviews were carried out with caregivers of children with nodding syndrome in Atanga sub county of Pader district; an area which had the majority of children affected by nodding syndrome in northern Uganda. Five focus groups were conducted, four of which were composed of only female caregivers and one group composed of only male caregivers. Participants in the groups were purposively selected to include only those taking care of a child or children with nodding syndrome. The participants for the discussions were recruited with the assistance of the nodding syndrome focal person based at Atanga Health Center III; one of the five designated nodding syndrome treatment centers in the region. The health center keeps up to date clinical records of all households that were affected by nodding syndrome in the designated area of supervision. Using these records, health workers at the center contacted the caregivers to invite them to participate in the focus group and in-depth discussions. Five in-depth interviews were carried out with caregivers who had more than one child affected with nodding syndrome. We interviewed a total of 54 caregivers who had a total of 54 children affected by Nodding syndrome. The majority of the participants were females (*n* = 45). There were only nine males. The oldest caregiver was 62 years and the youngest was 25 years. The oldest affected child for these caregivers was 18 years and the youngest affected child was 6 years of age.

### Materials, data sources, process, data management and analysis

We formulated a case vignette describing a mother of a child with nodding syndrome and then created questions about the challenges of taking care of such a child based on the case vignette. The case vignette and the questions were pre-tested in Luo, the local language spoken in the region. It was discussed with the investigators and some of the participants until it was felt that the questions were appropriately tailored to the caregivers. In developing the vignette, we used the nodding syndrome diagnostic criteria that had been developed at the international meeting organized by the Uganda Ministry of Health [2]. In order to ensure the credibility and trustworthiness of the data collected, the case vignette and questions were then translated and back translated into the native Luo language. The focus group and in-depth interview moderators were trained in qualitative data collection methods and human subjects research ethics prior to the data collection exercise.

Interviews were audio taped, translated and transcribed. Translators who were different from the moderators were used to translate and then transcribe the data into English. Two participants from each focus group were invited to listen to the transcription from their focus group discussion to determine whether it was an accurate reflection of their discussion. The data was then read and re-read several times by the investigators. In order to carry out the analysis, we used the NVivo software package. This is a qualitative data management software package that is used in organizing qualitative data mainly in the fields of behavioral sciences. Using the NVivo software package for qualitative analysis, data was coded, organized and referenced into a collection of ideas, known as ‘parent node’ in NVivo. Further management of data, that was deemed to be subsets of the original parent nodes, was then referenced into a hierarchical manner from which the child nodes were formed. After reading and re-reading, through parent and child nodes and using text search, also known as ‘queries’ in NVivo, we cross examined the nodes and formulated themes related to caregiver burden.

### Ethical statement

We obtained approval for the study from the Makerere University College of Health Sciences, School of Public Health’s Internal Review Board (IRB) and the Uganda National Council of Science and Technology. With support from the national coordinator for nodding syndrome, we sought and received permission from the district administration to access demographic records of caregivers of children with nodding syndrome. Along with the registry, members of the Village Health Team (VHTs) helped to identify the caregivers of the children with nodding syndrome. VHTs approached caregivers and informed them about the study. Information regarding an agreed upon date, time and physical location for when and where the interviews were to take place was communicated. Individual verbal and written voluntary informed consent was sought from all the participants after the purpose of the meeting and the study were explained. Permission was also sought to audiotape participants’ voices during the interviews. All caregivers who were approached agreed to be involved. The focus group and in-depth interviews were conducted by trained research assistants who observed protocol for the protection of human research subjects. Psychosocial support was available for those who needed it. Table 1, socio-demographic variables of the caregivers. Table 2, socio demographic variables of children with nodding syndrome.

**Table 1:** Socio- demographic variables for the nodding syndrome burden of care respondents

Age	Oldest	62	*N* = 54
	Youngest	25	
	Mean	36 (SD = 9.79)	
Religion		Male	Female
	Catholic	4	28
	Protestant	1	12
	Christian-Born Again	4	5
	Traditional Religion	-	-
Education Level	No Education	5	32
	1 – 7 yrs	4	13
	8 – 11 yrs	-	-
Marital status	Married	9	23
	Widowed	-	9
	Separated	-	8
	Single	-	5
Occupation	Peasant Farmer	9	45
	Formal Employment	-	-
Relationship to child	Biological Parents	8	38
	Other relatives including grandparents	1	7

**Table 2:** Socio demographic variables for children with nodding syndrome

Variable	Sub county 1 (Atanga)	
Age: mean Oldest Youngest	14.16 (2.4) 18 6	SD 9.7
Child gender (*n* = 54)	*n* (%)	
Male	25 (46.3)	
Female	29 (53.7)	
Class attended (*n* = 54)	*n* (%)	
Lower primary	8 (18.6)	
Middle primary	4 (7.5)	
Upper primary	1 (1.9)	
Not in School	38 (70.4)	
Religion of the child (*n* = 54)	*n* (%)	
Protestant	10 (18.5)	
Catholic	30 (55.6)	
Christian saved sect-	8 (14.8)	
Believers in tradition	0	
Not religious	6 (11.1)	
Is your father alive: (*n* = 54)	*n* (%)	
No	14 (25.9)	
Yes	40 (74.1)	
Is your mother alive: (*n* = 54)	*n* (%)	
No	2 (3.7)	
Yes	52 (96.3)	
Whom did you leave with: (*n* = 54)	*n* (%)	
With parent(s) or guardian	43 (79.6)	
With other relatives	4 (7.4)	
With grandmother or grand father	6 (11.1)	
Other persons	1 (1.9)	
School Attendance: (*n* = 54)	*n* (%)	
Going to school	7 (12.9)	
Not going to school	47 (87.1)	

## Results

What are the challenges of taking care of a child with nodding syndrome? As a consequence of caring for a child with nodding syndrome, key emerging themes included child safety concerns, burnout, social isolation and rejection, homicidal ideation, and physical and financial constraints.

### Theme 1: Child and caregiver safety concerns

Among caregivers of children with nodding syndrome, there is constant worry about the child’s safety. Participants reported that children sometimes wander away during the day when most caregivers have gone to work and the child has been left alone at home. This makes caregivers worried that the child or children could be harmed physically or emotionally. Participants reported the following:

“One is always on tension because the child can wander away to the bush, never to come back! I lost one of my children! He was among the first children who were affected in this community. My boy disappeared, he was found dead after two weeks, with a stench. He had wandered and died in the bush…” (Respondent in an In-depth interview)

Later on at night, a caregiver may lack sleep because he or she worries that the child might wake up unheard and wander away. If it is during the rainy season, then the child could easily drown:

“And during the rainy season she (caregiver) will be worried that the child might leave the house in the night and drown in the rainy puddles…” (Respondent 2 in Focus Group Discussion (FGD)2)

Wandering of the child is particularly worrisome, not only because of the constant fear that a child affected by nodding syndrome will verbally be attacked by members of the community, but in many instances, they are in danger of being physically attacked by members of the community who think that they (children) are not behaving appropriately. Participants reported that it is common that neighbors without affected children become verbally and physically abusive to children that are affected, as the quote below depicts:

“Sometimes there are some families that don’t have this illness and when your sick child gets an attack on their compound, they will start throwing unnecessary insults…”

“And there are others in the community who will pick a stick and beat a child found walking or wandering…”-an indication that they (the affected children) are unwanted in the neighborhoods” (Respondent 6 in FGD 2)

Not only are parents concerned and worried about their children’s wellbeing but they are also worried about their own safety. The children affected by nodding syndrome will commonly develop cognitive and behavioral problems. Due to poor judgment, they may wander and take food that does not belong to them, in which case they may be thought of as thieves. They may also become aggressive as a result of the syndrome, as is indicated in the quotation below:

“…right now my child just wanders off and he can go and pick food from anyone’s home. When I am not around and he is hungry, he will go and steal food. My child was never a thief. And he has now become a thief and is aggressive! He has become so aggressive that he can easily beat me since he is now 16 years old…the second one is 9 years old. And whatever he steals then the owner comes to ask me to pay for it, and if I don’t have money, then I have to be beaten as a punishment for having a child who is a thief!” (Respondent 7 in FGD 3)

Having a child that is healthy is every parent’s wish. The parents of children with nodding syndrome go through the emotional agony of seeing their children debilitated. Many of them commented on having shattered dreams in relation to their children’s futures:

“In life our children are our fruits for harvesting in old age and so if my child has no future; it means I also have no future…” (Respondent 5 in an in-depth interview)

### Theme 2: Burnout

Respondents reported that they felt overwhelmed by their children’s ill-health and in some instances this led to some caregivers abandoning their homes and children all together.

“I have gone through so much pain that I cannot go on to discuss more, I beg to stop here!” he bent his down and paused for a long period – then, raising his head up and looking straight at a tree in the compound said “it’s a bitter experience, really bitter” (Respondent in an in depth interview)

Often times, in dealing with burnout, a spouse has abandoned his or her matrimonial home and started another family leaving the affected children with their father or mother as the quotation illustrates:

“I know of a woman who on seeing that two of her children had the disease, she migrated to Gulu (another district), started afresh and married another man” (Respondent in an in depth interview)

“…some men have left their wives as a result of this illness. So we are now managing our sadness alone” (Female respondent in an in depth interview)

### Theme 3: Social isolation and rejection

Participants reported that taking care of a child with nodding syndrome is stigmatizing to both the child and the caregiver, and leads to social isolation and rejection by both the spouse and community members.

“With this illness, relations change. If in the past, many people were your friends, when a child develops this illness, people start avoiding you! Once you have a sick child, people begin to fear and stop visiting you. I am going through this because my home is close to the main road that everyone in the village uses… We were also told that we should have a separate cup and plate for the sick child. One day while at home, I overheard people saying that they could not come into my home for shelter from the rain because I have sick children! The social relations have completely broken down because of this illness. The person with a sick child might still want to stay with the other community members with healthy children. However, those with healthy children are afraid to stay with her/him. Your relatives will come to your neighborhood but will not come to your house!” (Respondent from FGD 3)

Although the neighbors and communities may not be discriminatory against children and families that are affected by nodding syndrome, the families affected by nodding syndrome may in turn, out of fear of rejection, keep to themselves, as this is illustrated in these quotes:

“….and you as well become afraid to go and visit them [neighbours]” (Female respondent 3 in Focus Group 1)

“I remember when my co-wife gave birth to a newborn, I went to visit them, but people said that I had an illness in my house, which would affect the newborn child. So I had to spit on the child as a way of preventing the child from getting afflicted with the illness in the future. So since that time I decided that I would rather stay with my children and stop visiting other people!” (Female respondent 1 in FGD 2)

“My child used to attend school without any problems and now that he is sick, the children are running away from him. Even if they are at home and the sick one is trying to join his friends to play, the other children run away from him. He is chased away because others think that he might spread his disease to the other children..” (Female respondent 3 in FGD 1)

Care giving can be a lonely journey and a source of agony for many caregivers. One woman with twins, both affected with nodding syndrome reported:

“I struggle alone. I always have to carry my twins and go to the garden, and as for my husband; he never comes with me to the garden…” (Respondent in an In depth Interview)

Another woman reported rejection by her husband because their children have nodding syndrome. Before the children were affected, her husband was frequently referred to by the community using their children’s names. However, when the children became afflicted, they were shunned by the husband and as a result, the people in the community stopped calling the woman’s husband by her children’s names. This created a sense of rejection and abandonment from both her husband and the community. The community currently uses other children’s names when referring to him- children that do not belong to the woman but to her co-wives. This is illustrated below:

“My husband with whom I have a child rejected my child completely! It is now three years! He said that he does not want, and that he will not waste his money on my sick child since there is no cure. I became very sad, because I would see him taking good care of the other children he has with the other woman; my co-wife. These other children do not have nodding syndrome! The community has even changed in the way they refer to him. Previously they would refer to him as the father of my child. Now they call him the name of the other woman’s child; my co-wife. He goes hunting and comes back with meat, but he will not share it with my household! He has rejected my children and I am struggling alone with them! (Female respondent 4 in FGD 1)

“Other children are going to school, but the sick one that you have cannot go to school. Others might already be in primary six but the sickness that your child has can prevent him or her from going to school.”

(Female respondent 5 in FGD 2)

### Theme 4: Homicidal and suicidal ideations

Homicidal ideations were commonly discussed among the caregivers. Participants reported that they saw death as an option with some reporting homicidal ideations while others reported suicidal ideations.

“I went to the garden, and when I returned, I found that my sick child had left home. People told me that I should quickly go after him, otherwise my sick child was about to reach a water source and was in danger of drowning! So I left and started running…I am not sure of what happened, but I fell down and got up crying, and I said to myself “if he died, maybe I would be at peace!” (Female respondent in FGD 2)

Suicidal ideations were also implied in various ways by some of the respondents as is illustrated below:

“My daughter who had this child with nodding syndrome is also sick but she remarried and is still having more children with that man. So I was thinking that I would be better off dead, so that I don't see the problems that my daughter is going through.” (Respondent 4 from FGD3)

“I thought about it for about five years! I didn't want to live and see what my child was going through. Whenever my child goes to people’s homes, he is chased away because people think that he might spread the illness…” (Respondent 5 in FGD3)

“For example it happened to some woman. She had three sick children and one day when they were having their meals, all the children ‘got an attack of the illness at the same time’. Their mother stayed with her food in her hands and didn't eat. On that day she [the mother] said that if she had any poison, she would have committed suicide..”(Respondent 8 in FGD 3)

“When you are sad, what comes to your mind right away is to commit suicide because the kind of life you are leading is so difficult that death becomes the immediate thought…” (Respondent 5 in FGD 3)

### Theme 5: Other psychological issues as a consequence of taking care of a child with nodding syndrome

Most caregivers also reported thinking too much, sadness and irritability, substance abuse and domestic violence, isolation, poor concentration, poor sleep, and weight loss.

“If there is an illness in the house, you are always quarrelling in the house…then one will inevitably feel sad” (Respondent 4 in FGD 2)

“If Apio (caregiver in focus group discussion guide) were invited to attend a party she would refuse to come because she doesn’t have that energy to come and interact with people. She wouldn't even think of staying with people” (Respondent 3 in FGD 2)

“I was so angry and I stopped socializing with other people because I have three sick children in my household”. (Respondent in In-depth Interview)

Many caregivers reported preoccupation with their children’s illness:

“She is thinking too much, she is thinking all the time”! in reference to caregiver in case vignette. (Respondent 6 in FGD 3)

“She is always thinking about her child and even when she is working in the garden, she is physically in the garden but her heart is at home with the sick child…” (in reference to caregiver in case vignette) (Respondent 1 in FGD 2)

“Sometimes I think I have an illness of thoughts…” (Caregiver 7 in FGD 3)

Caregivers reported feeling sad and their sadness was portrayed in various ways:

“When I start thinking of my child I feel an emptiness in my heart and if the child gets an attack at the time of eating, I lose my appetite.” (Respondent 5 in FGD 1)

“Sometimes I feel like my heart is bleeding.” (In depth interview respondent with 5 children affected by nodding syndrome)

Sleep disturbances were also described as plaguing the caregivers

“..if you have problems you cannot sleep. And many problems will come in addition to the problems you are already facing. Things like headache, thinking too much, hunger (loss of appetite).” (Respondent 3 in FGD 4)

### Theme 6: Physical and financial constraints

In the absence of healthcare services or support groups that relieve the caregivers of the burden of fulltime care giving, taking care of a child with nodding syndrome by the parents or guardians is always done at the expense of other activities that could help support the home:

“Having a sick child prevents one from going to the garden. So one may not have food in the house!” (Respondent 2 in FGD 4)

“One is always taking care of her child and may not find time to do other work to support her family” (Respondent 9 in FGD 1)

When there is scarcity of food and other resources, a caregiver may have to make the agonizing decision of catering for the needs of the healthy children and abandon the child with nodding syndrome as is illustrated below:

“There are some people who abandon those children and they may not even give them food because they say that those children are useless.” (Respondent 2 in FGD 4)

“Parents perceive spending money on the affected children as a wastage of resources. If there is no improvement in the children’s condition, then the parents do not see the point in continuing to spend the money on these children.” (Respondent 3 in FGD 3)

The experience of care giving is physically draining that in some instances, caregivers may not have the energy or motivation to do anything else for survival. The quote below illustrates this theme:

One problem that the caregiver has is that she is weak and she cannot even carry her child to the health center because she doesn’t have energy! (Caregiver 1 in FGD 1)

Not only is care giving so physically and emotionally draining, that taking on a care giving role implies that one is going to do less of what they used to do before the care giving role began. Economically the caregiver and the rest of the family suffers a financial setback. This was alluded to by participants in the focus groups:

“There may be other children that are difficult to take care of now because of the sick child, and their ability to attend school may also be compromised by their sick sibling” (Respondent 8 in FGD 4)

## Discussion

We explored the challenges that caregivers of children with nodding syndrome experience on a typical day in post conflict northern Uganda. Our findings reveal that a high burden of care was endorsed by most caregivers, with emotional agony as the most prominent theme capturing the care giving experience. Other themes reflecting the burden of care giving were subjective burden (burn-out, suicidal and homicidal ideations) as well as objective burden (child and caregiver safety concerns, social isolation and rejection, material and financial constraints).

Our study is the first of its kind in exploring caregiver burden specific to nodding syndrome. Therefore, the literature for the discussion is based on other diseases that carry a high burden of stigma and social exclusion and, on the caregiver burden of chronic diseases that compare in magnitude to nodding syndrome. The caregivers in this study reported emotional agony or distress as the most common theme representing the care giving experience with children affected by nodding syndrome. Some authors consider emotional burden that impacts on mental and emotional wellbeing as a subjective dimension of care giving [37]. With respect to subjective burden, caregivers also reported burn-out, suicidal and homicidal ideations, Caregivers suffer constant physical and emotional agony, social isolation and rejection. Nodding syndrome is a rare disease that is not well understood and because the disease manifests with neurological and psychiatric symptoms, it is understandably stigmatizing leading to social exclusion. This has been attested to in a publication on the functions of social exclusion in other stigmatizing illnesses [38]. Parents and guardians describe the experience of caring for a child with nodding syndrome as a lonely journey Whereas the social isolation and rejection might lead to a perception of fear and mistrust in those caring for affected children, the fear for the affected child’s safety has a genuine basis; children are frequently attacked by community members when they wander away from home as was reported in our results. Previous literature points out that persons perceived to have mental illness may be more at risk of being physically abused, than the general population may be at risk of being attacked by the mentally ill [39].

Regarding objective burden of care giving, our participants endorsed themes of child and caregiver safety concerns, social isolation and rejection, material and financial constraints. In keeping with the literature, child and caregiver safety concerns represent symptom-specific burden, material and financial constraints represent financial burden of caregiving and social isolation and rejection represent social burden of care giving [40]. In keeping with the extant literature, more females than males were caregivers and a primary biological family member was the most common caregiver [41]. Females traditionally take on caregiving roles which may be related to a more universal feature of care giving systems.

As individuals age, they increasingly have to depend on younger people for their mobility and other activities of daily living. The caregivers of children with nodding syndrome are deprived of the opportunity to envisage their affected children ever supporting them in old age. In the absence of a formal social security system, which caters for the needs of the aged and disabled in Uganda and Sub-Saharan Africa, children are a long term investment which parents look up to in their old age. Parents wish and hope that they will be outlived by their children and that their children will be able to take care of them in old age [42]. As evidenced from the results, this is not the case for the parents whose children are suffering from the debilitating nodding syndrome. Instead of hope that they will be taken care of by their children when they become of age, the parents have shattered dreams [43]. This is especially true for parents who have more than one child afflicted by nodding syndrome.

The characteristic features of nodding syndrome are exacerbated by the fact that the disease can be chronic and some of the associated disabilities are permanent. In a secondary analysis of the predictors of caregiver burden, impaired functioning of the care-recipient predicted a high level of caregiver burden [44]. Human beings long to have consistent and predictable lives, and even with challenges, humans would like their lives to get back to normal. Therefore, it is not surprising that some parents as evidenced from the results feel trapped, and would wish for the agony of taking care of a child with nodding syndrome to end through homicide so that they can mourn and resume with their normal lives.

Literature on the relationship of burnout and depression has shown that there is a positive correlation between burnout and depression. In a study that was carried out in Finland, results indicated that depression severity increased as burnout levels increased [45]. In this study, caregivers reported lack of energy to continue taking care of the affected children. They also reported symptoms of increased sadness, thinking too much (an expression of depression in this particular culture) [46], suicidal and homicidal ideations. A study carried out in Japan on caregiver burden and depression reported a high prevalence of depression of 46 % of caregivers in the sample [47]. Depending on the severity of symptoms, caregivers’ health is often negatively impacted upon by the role of care giving [48].

Family care giving is a fulltime unpaid occupation that drains the physical and economic resources of the caregivers of children with nodding syndrome. It is done at the expense of other activities that would generate income for the caregiver, a finding that has been described in other caregiver populations [49]. It is not surprising that caregivers of affected children are struggling with choices between providing basic needs to unaffected children as opposed to those children with nodding syndrome, in a setting of meagre resources. This dilemma may come about because the caregivers realize that the affected children may never grow to realize their full potential in life.

## Study limitations

Due to the qualitative nature of the study, the study findings are not generalizable to the general population of caregivers of children with nodding syndrome. The study was based on caregivers’ accounts and therefore subject to potentially biased reporting. The respondents may not have described their experiences of care giving in full, for two reasons.

First, some of the caregivers were interviewed just before a planned intervention and may therefore have had difficulty describing their care giving relationship with their child, especially if this had influenced symptoms or precipitated their seeking treatment. Second, the participants may not have wished to be reminded of the negative relational circumstances that may have led to their mental health symptoms, or emotional and behavioral problems in their children, and may therefore have evaded answering questions that were emotionally challenging. However considering that we sought to explore the lived experiences of the caregivers, the nature of the study design is the best that we could have employed for the results that we obtained from the participants. The strength of the study was that we analyzed themes to differentiate between various dimensions of caregiver burden.

### Implications

Caregivers of children with nodding syndrome describe negative physical, emotional and functional health challenges of long-term, informal care giving. They have important insights regarding those aspects of care giving that have negative influences on their health. Given the care giving themes emerging from our study, our study would therefore suggest that objective caregiver burden is comprised of those tasks required to care for the patient, whereas subjective caregiver burden indicates the extent to which the caregiver “minds” or is affected by performing these tasks.

Objective and subjective burdens for caregivers of children with nodding syndrome increases in resource poor settings with limited access to primary health care services and respite care such as post-war northern Uganda.

## Conclusion

We aimed to explore the experience of care giving in a scantily researched arena of nodding syndrome. Our findings point to a heavy burden of care in this portion of caregivers, with high levels of stigmatization and social exclusion, extreme psychological distress and physical exhaustion coupled with economic hardships. There is a need to explore the feasibility of integrating community-based psychosocial and mental health services into programming responses for caregivers and children affected by nodding syndrome.

Interventions that address these issues may have the potential to positively impact caregiver health and alleviate the distress and the stigma associated with nodding syndrome.
